# Mediterranean diet adherence is associated with antiviral, neuroimmune, and cardiometabolic proteomic profiles in people with HIV

**DOI:** 10.3389/fnut.2026.1801654

**Published:** 2026-04-22

**Authors:** Mónica Manzano, Elena Moreno, Laura Martín-Pedraza, Claudio Díaz-García, Santiago Moreno, Beatriz Navia, Sergio Serrano-Villar

**Affiliations:** 1 Department of Pharmacy and Nutrition, Faculty of Biomedical and Health Sciences, Universidad Europea de Madrid, Calle Tajo s/n, Villaviciosa de Odón, Madrid, Spain; 2 Department of Infectious Diseases, Hospital Universitario Ramón y Cajal, IRYCIS, Madrid, Spain; 3 CIBERINFEC, Instituto de Salud Carlos III, Madrid, Spain; 4 Department of Medicine, University of Alcalá de Henares, Guadalajara Campus, Alcalá de Henares, Spain; 5 Department of Nutrition and Food Science, Faculty of Pharmacy, Universidad Complutense de Madrid, Madrid, Spain; 6 Research Group VALORNUT-UCM (920030), Universidad Complutense de Madrid, Madrid, Spain; 7 Department of Medicine, Life Sciences Campus, Universidad Antonio de Nebrija, Madrid, Spain

**Keywords:** HIV, inflammation, Med-DQI, Mediterranean diet, proteomic signatures, TLR3, B4GALT1, MMP10

## Abstract

**Background:**

Dietary patterns can modulate the expression of proteins involved in inflammatory and metabolic pathways in people living with HIV. The aim of this study was to examine whether adherence to the Mediterranean diet is associated with distinct plasma proteomic signatures in PWH on stable antiretroviral therapy. Chronic inflammation persists despite virological suppression, making diet a potential modifiable factor in this setting.

**Methods:**

We included 25 adults with HIV on stable antiretroviral therapy. We assessed dietary intake using a 3-day dietary record, and adherence to the Mediterranean diet was evaluated using the Mediterranean diet Quality Index (Med-DQI). We quantified plasma protein expression using Olink proximity extension assay technology.

**Results:**

We found that participants with higher adherence to the Mediterranean diet (Med-DQI < 6) showed significantly higher expression of proteins involved in antiviral and mucosal immunity, neuroprotection, and metabolic regulation, including TLR3, JCHAIN, IL18R1, and NTF3. Conversely, lower adherence (Med-DQI ≥ 6) was associated with increased expression of proinflammatory and metabolically adverse proteins such as MMP10 and B4GALT1.

**Discussion:**

Our study indicates that healthier dietary patterns are associated with higher expression of proteins linked to antiviral, mucosal, metabolic, and neuroimmune pathways, and with lower expression of proteins previously related to inflammatory or adverse cardiometabolic profiles in people living with HIV. These differences in proteomic signatures may reflect diet-related variation in immune and metabolic pathways, although their clinical implications require further investigation.

## 1 Introduction

Persistent immune dysregulation and chronic inflammation remain prevalent in people living with HIV (PWH) despite effective antiretroviral therapy (ART) (1–5). Proteomic studies have described immune and metabolic alterations in PWH; however, the role of modifiable factors such as dietary patterns has received little attention. In the general population, proteomic analyses link dietary patterns to distinct plasma protein profiles involved in inflammatory and metabolic pathways. Evidence from large-scale proteomic and nutritional studies shows that healthy dietary patterns, particularly the Mediterranean diet, associate with lower systemic inflammation and more favorable cardiometabolic profiles (6–9). Because plasma proteins integrate signals from antiviral immune activity, mucosal barrier function, and metabolic regulation, dietary exposures could influence biological pathways that remain dysregulated in PWH despite ART. Given that plasma proteins capture key biological processes related to antiviral responses, mucosal immunity, and cardiometabolic regulation, we hypothesized that adherence to the Mediterranean diet would be associated with distinct plasma proteomic profiles in PWH receiving stable ART.

## 2 Materials and methods

### 2.1 Study design and sample collection

We conducted a cross-sectional study with 25 participants recruited from the HIV unit at Hospital Universitario Ramón y Cajal in Madrid, Spain. Among the participants, 2 were women. The inclusion criteria were confirmed HIV infection, age 18 years or older, and the use of ART with undetectable plasma HIV RNA (<40 copies/ml) for at least 48 weeks. Patients with acute or intercurrent health problems were excluded.

The Ethics Committee of Hospital Ramón y Cajal (Ref. 030/20) approved the protocol, and all participants provided informed consent. Dietary questionnaires and blood samples were collected on the same day.

### 2.2 Dietary data

A three-day dietary record, including two weekdays and one weekend day, was used to collect all food, beverages, and food supplements consumed by the participants. To ensure accurate completion of the questionnaire, participants received detailed written instructions. These instructions covered food and dish ingredients (where possible), cooking methods, brand names of products, and quantities, which could be recorded using household measurements. Additionally, the research group’s nutritionist reviewed all data to minimize errors, such as identifying unrealistic servings, fluid intake, or any other mistakes.

We processed the dietary information using DIAL software version 3.0.0.12 (Alce Ingeniería, Madrid, Spain), based on data from the Spanish Food Composition Tables (10). This resulted in tables containing energy intake, caloric profiles of macronutrients, vitamin and mineral intake, and the intake variables of 14 food groups measured in grams per day (10, 11).

### 2.3 Dietary quality index (Med-DQI)

To assess diet quality, we used the Med-DQI dietary quality index, which is regarded as one of the most appropriate indexes for evaluating the quality of the Mediterranean diet in adults (12–14). The components assessed included the percentage of saturated fatty acids relative to total energy, cholesterol (mg), grams of meat, mL of olive oil, grams of fish, and grams of fruits and vegetables. Each nutrient or food group was assigned a score of 0, 1, or 2 based on recommended guidelines. Med-DQI scores of 1–4 indicate good diet quality, 5–7 indicate moderately good diet quality, 8–10 indicate moderately poor diet quality, and 11–14 indicate poor diet quality. In the present study, the median Med-DQI value of the sample was 6. Participants were categorized according to this cutoff into higher adherence (HMD) and lower adherence (LMD) groups.

### 2.4 Measurements in blood samples

We collected fasting venous blood samples in EDTA tubes and immediately processed them to obtain plasma, which we aliquoted and stored at −80 °C until analysis. In total, we worked with 25 EDTA plasma aliquots (one sample per participant), which we thawed and vortex-mixed before loading onto the Olink® plates.

We performed proteomic profiling using the Olink® Inflammation panel, which quantifies 368 inflammation-related proteins based on Proximity Extension Assay (PEA) technology. Of the 25 samples, 100% passed the internal quality control (QC) procedures, and 338 of the 345 proteins that remained after QC filtering were detected in more than 50% of the samples. We considered proteins non-detected when their expression levels were below the limit of detection in more than 50% of samples in both groups, and we excluded them from further analyses.

To minimize batch effects, all baseline samples were analyzed on a single Olink assay plate under identical experimental conditions. Protein concentrations were expressed as Normalized Protein eXpression (NPX) values, a relative quantification unit on a log2 scale provided by the Olink system.

We performed all assays according to the manufacturer’s instructions (15).

### 2.5 Statistical analysis

Numerical variables are presented as mean ± standard deviation (SD) or median (25th–75th percentile), depending on their distribution, and categorical variables as absolute frequencies and percentages. Normality was assessed using the Shapiro–Wilk test. Between-group comparisons were performed using the Mann–Whitney U test for numerical variables and the Chi-square (χ^2^) test for categorical variables. Statistical significance was set at *p* < 0.05, with false discovery rate correction applied using the Benjamini–Hochberg method (qFDR < 0.10).

Given the high dimensionality of the proteomic dataset (338 proteins), we applied Sparse Partial Least Squares Discriminant Analysis (sPLS-DA) using the mixOmics R package to characterize the separation between participants with higher adherence to the Mediterranean diet (HMD) and those with lower adherence (LMD), as defined by the median Med-DQI score. The model was fitted using two components, and variable loadings were examined to identify proteins contributing most strongly to group discrimination.

To focus on the most informative features, we selected the proteins with the highest absolute loadings from the sPLS-DA model, corresponding to those most strongly associated with each dietary-adherence group. These proteins were subsequently subjected to independent statistical validation using the Mann–Whitney U test.

To evaluate whether these associations could be influenced by demographic or clinical factors, we conducted exploratory adjusted regression analyses for the proteins showing significant differences between dietary-adherence groups. For each protein, we fitted linear or robust regression models including Med-DQI as the main predictor and the following covariates: body mass index (BMI), CD4 + T-cell count, and ethnicity. ART regimen was not included because all participants were receiving antiretroviral therapy, resulting in no variability.

All statistical analyses were performed using IBM SPSS Statistics Version 20.0 (Armonk, NY, USA) and R software (version 4.1.2; R Core Team, Vienna, Austria), including the mixOmics package for sPLS-DA (16, 17).

## 3 Results

### 3.1 Description of the general population

We analyzed data from 25 adults living with HIV, 12 of whom were classified in the “Higher Mediterranean diet adherence” (HMD) group and 13 in the “Lower Mediterranean diet adherence” (LMD). The mean age was 48.6 years, and the majority of participants were of Caucasian ethnicity. All participants were receiving ART, and the mean BMI was 24.4 kg/m^2^. A full description of the study population is provided in Table 1.

**Table 1:** Description of the study population.

Characteristics	Higher adherence MD (*n* = 12)	Lower adherence MD (*n* = 13)	Total (*n* = 25)	*p*-value
Age, mean ± DE, years^(*t*)^	46.4 ± 10.8	50.6 ± 11.3	48.6 ± 11.0	0.353
Sex, *n* (%)^(*χ*2)^				0.953
Female	1 (7.7%)	1 (8.3%)	2 (8.0%)	
Male	12 (92.3%)	11 (91.7%)	23 (92.0%)	
Ethnicity, *n* (%)^(*χ*2)^				0.056
Caucasian	12 (92.3%)	8 (66.7%)	20 (80.0%)	
Latin American	0 (0.0%)	4 (33.3%)	4 (16.0%)	
Sub-Saharan African	1 (7.7%)	0 (0.0%)	1 (4.0%)	
BMI (kg/m^2^), median (P25-P75)^(*t*)^	24.5 (23.4–26.9)	24.4 (22.2–26.6)	24.4 (23.1–26.6)	0.413
CD4, median (P25-P75), cels/uL^(*U*)^	571.3 (454.9–807.8)	531.7 (435.8–768.1)	533.4 (455.4–768.1)	0.957
ART, *n* (%)	12 (100%)	13 (100%)	25 (100%)	–
Undetectable HIV RNA, *n* (%)	12 (100%)	13 (100%)	25 (100%)	–

Results are presented as mean ± SD or median (25th–75th percentile) for continuous variables, and as *n* (%) for categorical variables. Significant differences between adherence groups (*p* < 0.05). (*t*): *p*-value calculated using the independent samples *t*-test; (*U*): *p*-value calculated using the Mann–Whitney *U*-test; (*χ*^2^): *p*-value calculated using the Chi-square test. BMI, Body Mass Index; ART, Antiretroviral therapy.

### 3.2 Proteomic signatures associated with Mediterranean diet adherence (Med-DQI)

We conducted an sPLS-DA to assess differences in protein expression between participants with HMD (Med-DQI < 6) and those with LMD (Med-DQI ≥ 6), analyzing a total of 338 proteins after passing the quality control procedures. We optimized model complexity using internal cross-validation (5-fold, 50 repetitions) and selected a two-component model. We evaluated model performance using repeated stratified cross-validation with *centroids.dist* as the prediction distance and by tuning the sparsity parameter (*keepX*). The median balanced error rate (BER) was approximately 0.55 and the overall error rate 0.54, reflecting modest discriminative performance consistent with the small sample size (n = 25) and the biological complexity of the phenotype.

Despite the modest overall performance, variable selection was stable for Component 1. The first component accounted for 12% of the variance in X and yielded a consistent set of six proteins with selection stability ≥ 0.80 across resampling: TLR3, JCHAIN, IL18R1, NTF3, MMP10 and B4GALT1. Component 2 (25% of the variance) showed low selection stability, and therefore biological interpretation focused on Component 1 to minimize overfitting.

**Figure 1:**
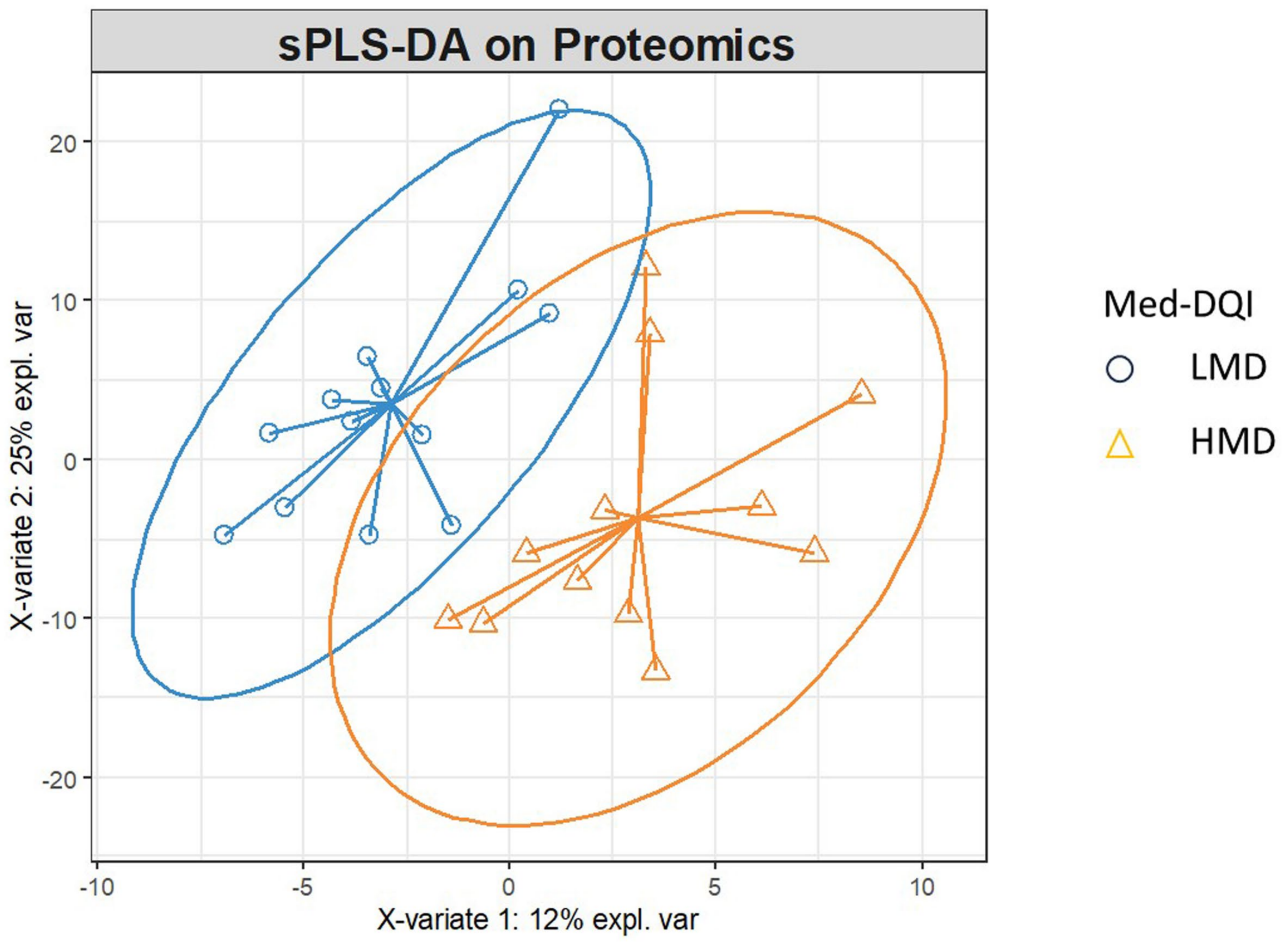
Sample plot–confidence ellipses: Samples of higher adherence Mediterranean diet (Med-DQI < 6) and lower adherence Mediterranean diet (Med-DQI ≥ 6) form different clusters, indicating differences in diet.

**Figure 2:**
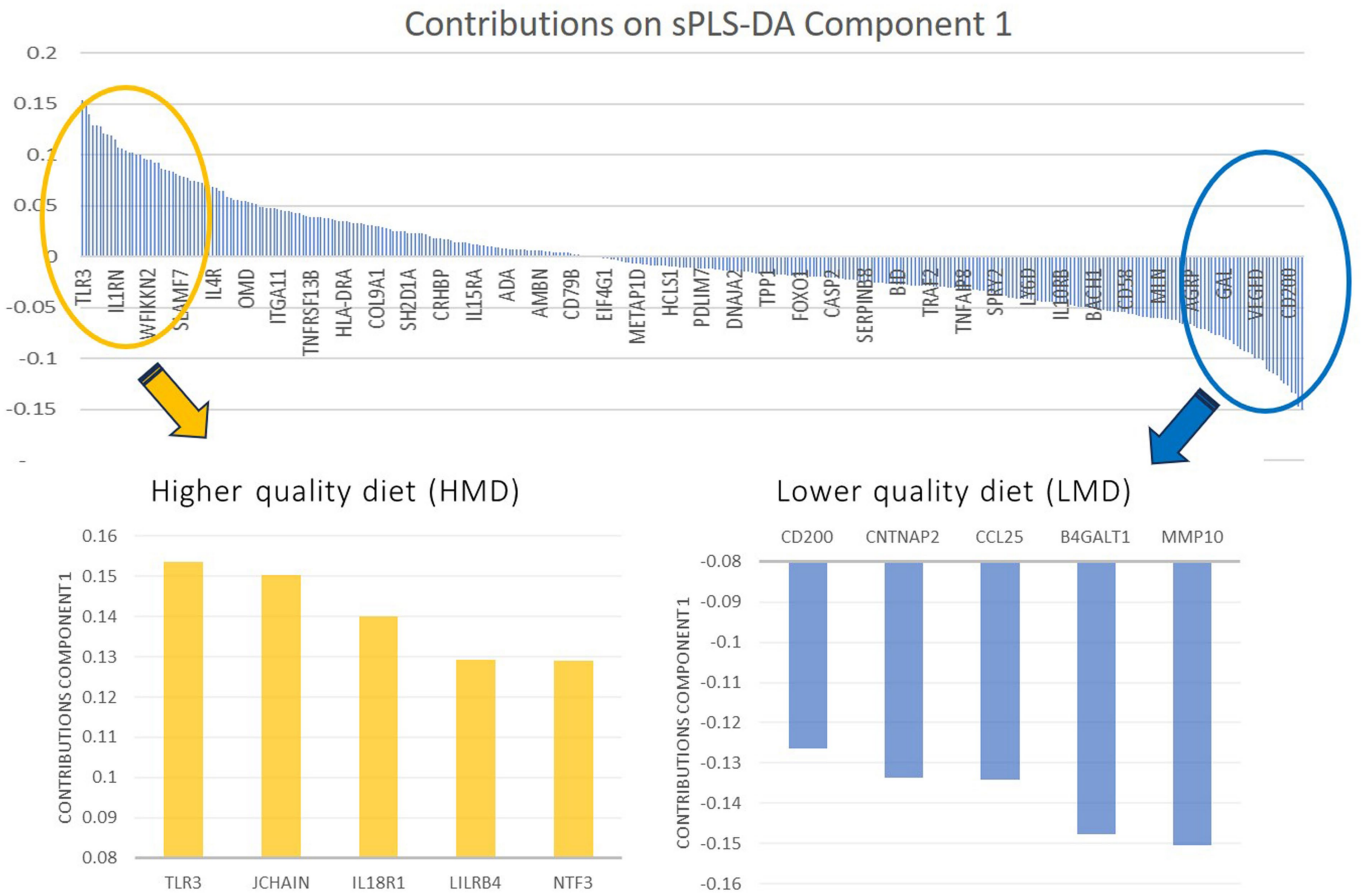
Contributions on component 1 (sPLS-DA 1): Constructed by the loading vector, it shows the most relevant proteins differentially abundant in the higher quality diet group compared to the lower quality diet group. Due to the high number of proteins analyzed, we extracted the 5 most relevant proteins that contribute to each Med-DQI adherence group.

As shown in Figure 1, the sPLS-DA separated both groups with minimal overlap between confidence ellipses, with Component 1 and Component 2 explaining 12 and 25% of the variance, respectively. Figure 2 shows the proteins that contributed most to this separation. Given the large number of proteins analyzed, we selected the 10 most relevant proteins overall, corresponding to the five most discriminant proteins in each diet-quality group, to highlight the features that most strongly contributed to group separation. Proteins with higher expression in the HMD group included TLR3, JCHAIN, IL18R1, LILRB4, and NTF3, whereas MMP10, CCL25, CNTNAP2, CD200, and B4GALT1 showed higher expression in the LMD group.

### 3.3 Key proteins differentiating HMD vs. LMD

**Figure 3:**
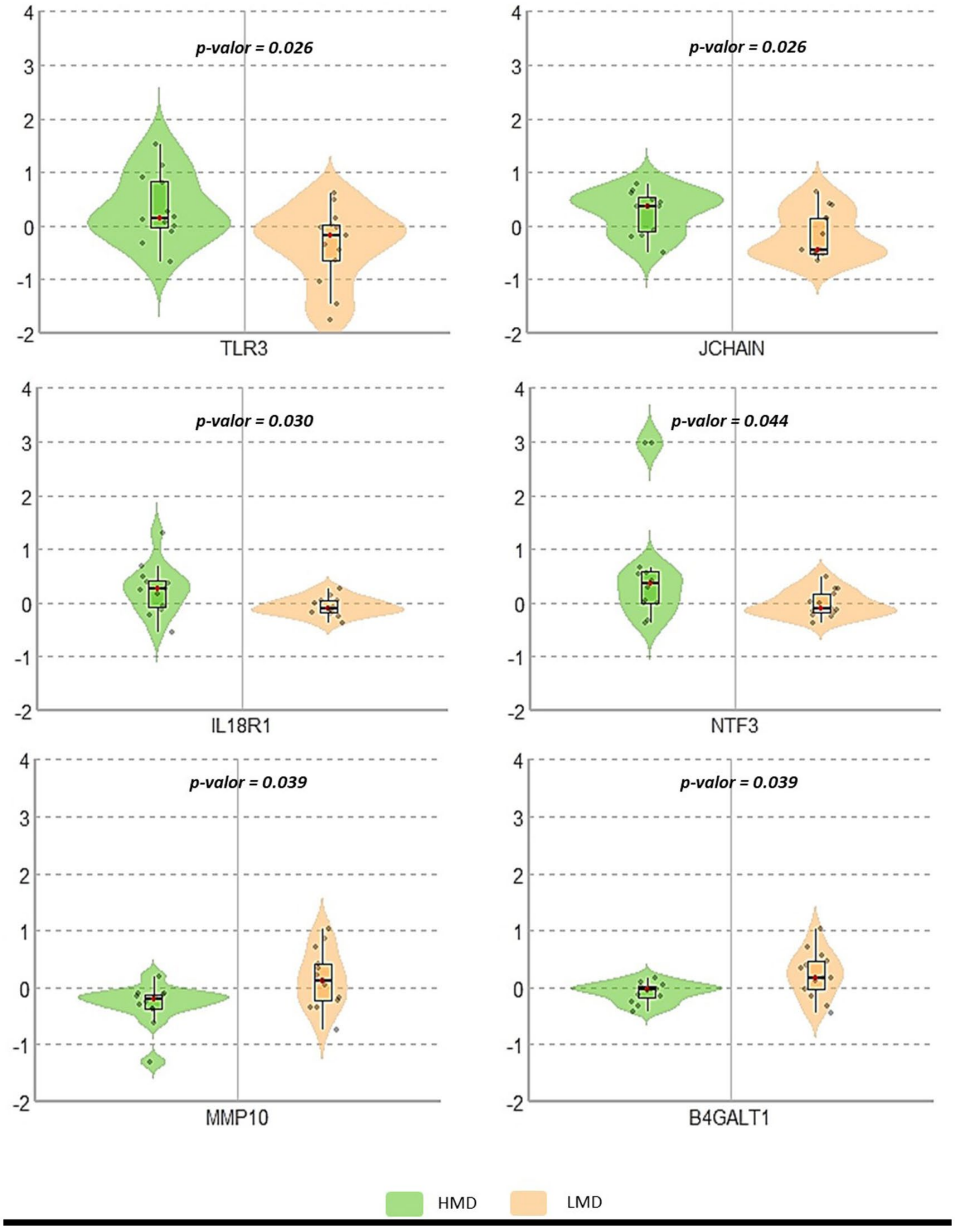
Normalized protein expression by Mediterranean diet adherence group: The graph shows the distribution of each protein with the median (black line) and individual points representing participant values. Proteins with statistically significant differences (*p* < 0.05) between Mediterranean diet adherence groups are displayed. The Y-axis unit is expressed in NPX (normalized protein expression).

To enhance analytical robustness, we performed an additional statistical validation using the Mann–Whitney U test, with the corresponding results presented in Table 2, and visualized the distribution of protein levels as violin plots in Figure 3. Of the 10 proteins selected from the sPLS-DA model, six showed statistically significant differences between the HMD and LMD groups. Specifically, TLR3 (*p* = 0.026), JCHAIN (*p* = 0.026), IL18R1 (*p* = 0.030), and NTF3 (*p* = 0.044) exhibited higher expression in participants with better adherence (Med-DQI < 6), whereas MMP10 (*p* = 0.039) and B4GALT1 (*p* = 0.039) were more highly expressed in those with lower adherence (Med-DQI ≥ 6).

**Table 2:** Normalized protein expression by Mediterranean diet adherence group.

Proteins	Higher adherence MD (*n* = 12)	Lower adherence MD (*n* = 13)	Total (*n* = 25)	*p*-value
**TLR3**	**0.15 (−0.05–0.85)**	**−0.17 (−0.65–0.01)**	**0.00 (−0.36–0.27)**	**0.026**
**JCHAIN**	**0.37 (−0.13–0.55)**	**−0.44 (−0.53–0.14)**	**0.13 (−0.46–0.42)**	**0.026**
**IL18R1**	**0.26 (−0.09–0.44)**	**−0.10 (−0.18–0.04)**	**0.00 (−0.16–0.27)**	**0.030**
LILRB4	0.14 (−0.06–0.91)	0.01 (−0.10–0.07)	0.02 (−0.10–0.28)	0.157
**NTF3**	**0.36 (0.00–0.60)**	**−0.12 (−0.18–0.16)**	**0.01 (−0.16–0.42)**	**0.044**
CD200	−0.03 (−0.30–0.09)	0.01 (−0.05–0.46)	−0.01 (−0.18–0.23)	0.142
CNTNAP2	0.02 (−0.42–0.12)	0.35 (−0.06–0.81)	0.10 (−0.23–0.42)	0.073
CCL25	−0.28 (−0.81–0.16)	0.02 (−0.02–0.49)	0.02 (−0.58–0.26)	0.092
**B4GALT1**	**−0.04 (−0.21–0.02)**	**0.16 (−0.03–0.46)**	**0.01 (−0.15–0.18)**	**0.039**
**MMP10**	**−0.21 (−0.37– −0.12)**	**0.13 (−0.23–0.42)**	**−0.16 (−0.34–0.20)**	**0.039**

Results are presented as median (interquartile range). Significant differences between Higher and Lower Mediterranean diet adherence groups (*p* < 0.05), highlighted in bold, were calculated using the Mann–Whitney *U*-test.

After Benjamini–Hochberg correction within the sPLS-DA–selected subset, all six proteins remained significant (*q* < 0.10). When applying FDR correction across the full panel of 338 proteins, no proteins met the adjusted significance threshold, consistent with the limited statistical power of this exploratory dataset. The concordance between multivariate stability (Component 1) and univariate significance after FDR suggests a consistent analytical convergence, albeit in an exploratory context and with a small sample size.

BMI, CD4 count, and ethnicity did not differ significantly between the HMD and LMD groups (Table 1), suggesting that these variables were unlikely to confound the initial comparisons. To further evaluate whether these factors could act as potential confounders, we performed exploratory regression analyses restricted to the six proteins showing significant between-group differences. Each model included Med-DQI as the main predictor and was adjusted for BMI, CD4 + T-cell count, and ethnicity. ART regimen was not included because all participants were receiving antiretroviral therapy, resulting in no variability.

After adjustment, three proteins remained significantly associated with Med-DQI: TLR3, MMP10, and JCHAIN. For B4GALT1, IL18R1, and NTF3, the associations remained directionally consistent but did not reach statistical significance. BMI, CD4 count, and ethnicity were not significant predictors in any model, suggesting that the observed proteomic differences between adherence groups are unlikely to be explained by these potential confounders.

## 4 Discussion

The relationship between dietary patterns and protein expression is an emerging area of research that examines how specific dietary habits can modulate protein expression, thereby influencing susceptibility to disease. Current evidence indicates that diet not only provides essential substrates required for protein synthesis but also regulates proteins involved in key metabolic, inflammatory, and immune pathways (18–20).

In PWH, nutritional factors have been increasingly recognized as modulators of immune function and chronic inflammation even under effective antiretroviral therapy (21–23). Consistent with this framework, in our cohort higher adherence to the Mediterranean diet was associated with distinct plasma protein expression patterns related to antiviral defense, mucosal immunity, and cardiometabolic regulation.

In our study, we found that individuals with HMD exhibited higher expression levels of both TLR3 and JCHAIN compared to those in the LMD group. Reduced TLR3 expression has been reported in people living with HIV, and this receptor plays a key role in innate immunity through antiviral and immunomodulatory functions (24, 25). Increased JCHAIN expression supports the formation of IgA and IgM, which are involved in mucosal immune processes (26, 27). Therefore, the higher expression observed in the HMD group may reflect pathways associated with mucosal immune defense, although these findings should be interpreted as associative rather than indicative of enhanced mucosal protection.

In this analysis, we observed higher IL-18R1 expression in the HMD group. Although IL-18 is traditionally described as a pro-inflammatory cytokine, evidence indicates that IL-18 signaling is also involved in neuronal stability, intestinal barrier integrity, and metabolic homeostasis, beyond its classical immune functions (28–31). Deficient IL-18 signaling has been associated with obesity, dyslipidemia, insulin resistance, and mitochondrial dysfunction (32, 33). Accordingly, increased IL-18R1 expression observed in the HMD group may reflect pathways related to IL-18 signaling, which in other contexts participate in antiviral responses, barrier integrity, and metabolic functions. These findings should be interpreted as associative rather than indicative of functional consequences.

In our study, PWH with HMD showed higher expression of NTF3 compared to those in the LMD group. NTF3 is a neurotrophin involved in neuronal survival, development, and regeneration, and contributes to synaptic plasticity and cognitive function (34–36). Higher NTF3 expression in individuals with greater dietary adherence may relate to pathways involved in neuronal maintenance, although no functional neuroprotective effect can be inferred. These observations are consistent with evidence that adherence to the Mediterranean diet may be associated with pathways relevant to cognitive and neurological function in PWH (37, 38).

In contrast, PWH with lower adherence to the Mediterranean diet showed higher expression of MMP10 and B4GALT1.

MMP10 emerged as the most highly expressed protein in this group. MMP10 is involved in extracellular matrix remodeling and has been linked to chronic inflammation and disease progression, including cancer (39, 40). Higher MMP10 expression may be related to inflammatory pathways previously linked to cardiometabolic or oncologic processes, although these associations should be interpreted cautiously and do not imply increased risk in the absence of clinical outcomes (41–43).

Moreover, elevated MMP10 levels have been associated with atherosclerosis and cardiovascular dysfunction. Conversely, antioxidant-rich components and dietary fiber characteristic of the Mediterranean diet have been associated with reduced carotid intima–media thickness and lower cardiovascular risk (44, 45). Previous studies have also reported associations between MMP10 overexpression and lymph node metastasis in certain cancers, which should be interpreted as associative observations (46, 47).

B4GALT1 is a key enzyme in glycan biosynthesis and plays an important role in cellular signaling and immune regulation. Dysregulated B4GALT1 expression has been reported in acute myeloid leukemia and other malignancies, as well as in metabolic and neurodegenerative disorders. Elevated B4GALT1 expression has been associated with aberrant glycosylation patterns involved in processes such as cellular invasion and tumor progression. In this context, higher B4GALT1 expression in the LMD group may reflect a biological context associated with chronic inflammation and a greater burden of comorbidity, including cancer-related complications, in PWH (48–51).

Nevertheless, within the current state of knowledge, our findings add to the evidence that dietary differences are associated with variability in inflammatory biomarkers and relevant biological pathways in people living with HIV. Lower adherence to the Mediterranean diet, associated with increased expression of proinflammatory proteins, may reflect a less favorable proteomic profile, although such associations should not be interpreted as indicators of clinical risk. Improving adherence to this dietary pattern may contribute to a more favorable proteomic profile and help mitigate the burden of chronic comorbidities in this population.

In conclusion, our findings indicate that adherence to the Mediterranean diet is associated with a more favorable proteomic profile in people with HIV, characterized by differences in the expression of proteins involved in pathways related to innate immune responses, mucosal processes, and metabolic and neurobiological regulation, including TLR3, JCHAIN, IL-18R1, and NTF3. In contrast, lower adherence was associated with increased expression of proteins involved in inflammatory processes, cardiovascular dysfunction, and adverse glycosylation pathways, such as MMP10 and B4GALT1. Overall, these results support diet quality as a modifiable factor influencing key immune and metabolic pathways in this population.

Our study has several limitations that should be considered. The small sample size (*n* = 25) limits the generalizability of the findings, and the cross-sectional design precludes causal inference between diet and protein expression. Dietary intake was assessed using short-term self-reported data, which may not fully reflect usual dietary patterns over longer periods. These constraints underscore the need for larger and more diverse cohorts, as well as longitudinal studies, to confirm these associations and further explore the potential of signatures linking diet and protein expression as biomarkers of immune function in PWH. Despite these limitations, our findings provide an initial framework for understanding how dietary patterns may relate to protein expression and immunometabolic variability in this population.

## Data Availability

The dataset generated and analyzed during this study is available in GitHub via the following link: https://github.com/einlabryc/Diet_HIV_proteomics.
